# Smoking among patients followed at the department of psychiatry

**DOI:** 10.1192/j.eurpsy.2021.1513

**Published:** 2021-08-13

**Authors:** A. Kerkeni, W. Abbes, M. Abbes, K. Medhaffer, K. Zitoun, L. Ghanmi

**Affiliations:** The Department Of Psychiatry, Hospital of gabes, Gabes, Tunisia

**Keywords:** dependence, Smoking

## Abstract

**Introduction:**

Smoking and nicotine dependence are particularly common in patients with a psychiatric disorder compared to the general population.

**Objectives:**

To study the prevalence of smoking in patients followed at the department of psychiatry and to assess their dependence on nicotine.

**Methods:**

This was a cross-sectional, descriptive and analytical study. The study focused on patients followed at the department of psychiatry of the regional hospital of Gabes. Sociodemographic and clinical data were assessed. Fagerstrom questionnaire in its validated French version was used to assess the nicotine dependence. Data were analyzed using the software SPSS (20th edition).

**Results:**

100 patients were included. They were male (60%) and single (50%) and with a mean age of 45.3 years [18-71]. The three most common pathologies were anxiety disorders (31%), schizophrenia (30%) and depression (29%). Among the patients surveyed 48% were smokers. Of which, 93.7% smoked cigarettes, 20.8% snorted chewing tobacco and 12.5% smoked hookah. The average number of pack-years was 11.6, with an average of 22.8 cigarettes per day. The mean duration of regular smoking was 19.1 years. Regarding nicotine dependence, 42% of patients were dependent. Regarding the degree of dependency, 43.7% were heavily dependent, 25% were weakly dependent and 18.8% were moderately dependent. Smoking was significantly associated with the male gender (p≤10-3), alcoholism (p=0.002) and schizophrenia (p=0.006).

**Conclusions:**

Results of our study show that smoking is frequent in patients followed at the psychiatry department. This aspect should be taken into account during the psychiatric evaluation, especially when therapeutic resistance occurs.

